# A Rare Adverse Event of Rhabdomyolysis Caused by Sacubitril/Valsartan

**DOI:** 10.3390/diseases7020038

**Published:** 2019-05-14

**Authors:** Prashanth Rawla, Jeffrey Pradeep Raj, Sajid Melvin George, Pavani Nathala, Anantha R. Vellipuram

**Affiliations:** 1 Department of Internal Medicine/Hospitalist, SOVAH Health, Martinsville, VA 24112, USA; 2 Department of Clinical Pharmacology, Seth G.S. Medical College & King Edward Memorial Hospital, Mumbai 400012, India; jpraj.m07@gmail.com; 3 Department of Medicine/Nephrology, SOVAH Health, Martinsville, VA 24112, USA; sajidmelvin@gmail.com; 4 Division of Infectious Diseases, University of Louisville, Louisville, KY 40202, USA; pavani.nathala@louisville.edu; 5 Department of Neurology, Texas Tech University Health Sciences Center, El Paso, TX 79905, USA; vellipuram@gmail.com

**Keywords:** rhabdomyolysis, entresto, sacubitril/valsartan, adverse drug reaction

## Abstract

Rhabdomyolysis is caused by extensive damage to skeletal muscles resulting in elevated creatine phosphokinase (CPK), Lactate dehydrogenase (LDH), and aspartate aminotransferase (AST), leading to life-threatening consequences like acute renal failure, cardiac arrhythmias, and hyperthermia. A variety of causes for muscle damage are known, and one of the most common is drug-induced. Statins and many other agents are known to induce muscle damage, but here we report Entresto™ (Sacubitril/Valsartan) induced rhabdomyolysis which has not been previously reported as solely responsible in the literature.

## 1. Introduction

Rhabdomyolysis is a clinical syndrome caused by extensive damage to skeletal muscles resulting in elevated creatine phosphokinase (CPK), Lactate dehydrogenase (LDH), and aspartate aminotransferase (AST), leading to life-threatening consequences like acute renal failure, cardiac arrhythmia, and hyperthermia [1]. The characteristic presentation of rhabdomyolysis is a triad comprised of muscle pain, weakness, and dark colored urine, though all three are not always present in a patient [2]. A variety of causes for muscle damage have been listed in the literature, some of which include trauma (crush injury, road traffic accident), heat (heat stroke, lightning strike, burns), severe muscle exertion (intense shivering, intense exercise), ischemic limb injury, metabolic disorders (hypothyroidism, diabetic ketoacidosis, electrolyte imbalances), genetic disorders (carnitine deficiency, McArdle’s disease, lactate dehydrogenase deficiency, Duchenne muscular dystrophy), viral and bacterial infections, inflammation (polymyositis, dermatomyositis, snake bites), and certain toxins and medications (statins, cyclosporine, erythromycin, colchicine, cocaine, amphetamines, ecstasy) [3]. However, almost three-fourths of the initial episodes of rhabdomyolysis are due to acquired causes and of those, the most common is prescription drug-induced rhabdomyolysis [4]. Here, we report a case of Entresto™ (Sacubitril/Valsartan) induced rhabdomyolysis which has not been reported as a solely inducing agent in the literature thus far. Entresto™ is a fixed-dose combination of sacubitril, a neprilysin inhibitor, and valsartan, an angiotensin II receptor blocker (ARB). It is indicated in patients with chronic heart failure (New York Heart Association [NYHA] class II-IV) with reduced ejection fraction, to decrease the risk of hospitalization for heart failure and cardiovascular death [5]. Though rhabdomyolysis is listed as a potential adverse drug reaction to Entresto™, there have been no case reports of Entresto™ as being the sole cause of severe rhabdomyolysis. Recently two case reports have reported a similar clinical scenario except for the fact that these patients were also on concomitant statin therapy [6,7]. However, we report a patient here who was not on concurrent statin therapy or any other drugs known to cause rhabdomyolysis.

## 2. Case Narrative

A 53-year old African-American woman presented to the emergency room (ER) with complaints of weakness for the past three days. The weakness was progressively worsening and was described as generalized and was associated with confusion for one day before presentation to the ER. The patient was living at home with her two daughters, and the patient reported that she had chills overnight. However, there was no history suggesting the involvement of respiratory, cardiovascular, and gastrointestinal symptoms. Her urine was dark in color, but there was no history of dysuria, pain, or foul smell while passing urine. The patient also had a past medical history significant for non-ischemic cardiomyopathy status post automatic implantable cardioverter defibrillator (AICD) implantation, systolic congestive cardiac failure, status post cardiac catheterization with an ejection fraction of 30–35%, three months before the current presentation. The patient also reported multiple other co-morbidities like tobacco abuse (smokes half a pack of cigarette a day—20 years), essential hypertension, type-2 diabetes mellitus, hyperlipidemia, fibromyalgia on chronic opiates, obesity, anxiety, chronic kidney disease (CKD) stage-3, anemia of CKD, Chronic obstructive pulmonary disease on home oxygen and gastro-esophageal reflux disease. Besides AICD placement and Cardiac Catheterization, she had a past surgical history for Esophagogastroduodenoscopy, colonoscopy, benign tumor removal of her foot, and some back surgery where the patient had placement of rods and pins in her spine. The family history was significant for diabetes mellitus and hypertension, and she also gave a history of allergy to Pregabalin.

On examination, the patient was lying calmly in the bed with no acute distress. Her vital signs examination revealed hyperthermia (99.8 F) and hypotension (blood pressure = 82/45 mm of mercury). She was maintaining a saturation of 97% on 2 L oxygen. Her mucous membranes were dry. She had minimal bilateral pedal edema up to the ankles. On central nervous system examination, the patient was conscious, oriented, and was able to follow commands. Systemic examination otherwise was normal. Blood investigations at admission revealed a normocytic normochromic anemia, leukocytosis with neutrophil predominance, mild hyponatremia, high anion gap metabolic acidosis, acute renal failure, hypocalcemia, severely elevated creatinine kinase, and a reversed albumin-globulin ratio. The details of all the blood investigations and their changes during the hospital course are reported in Table 1. A urine toxicology screen was positive for opioids and benzodiazepines. The findings were suggestive of rhabdomyolysis, and a revisit to the history looking in detail for any other potential causes for rhabdomyolysis revealed none except for the addition of Entresto™ to her existing medication regimen five days prior to presentation to the ER and a possible infection as the leucocyte count was elevated.

Meanwhile, the patient was started on intravenous fluids, infusion of vasopressors nor-epinephrine and dopamine which helped resolve the hypotension within few hours. She was also started on antibiotics, vancomycin, and piperacillin-tazobactam empirically for the suspected infection. Influenza was checked and was negative. Blood cultures were all negative. Since no source of infection was found, antibiotics were discontinued after a few days. Also, the leucocyte counts dropped back to normal the next day once the patient was rehydrated. Entresto™, diuretic bumetanide, amiodarone, and Coreg were stopped at admission. Her CPK levels and renal function slowly improved with supportive management. As the hypotension resolved within 2 days, all other medications except Entresto™ were restarted. Here, concomitant medications include Calcitriol capsules, ferrous sulfate tablets, fluticasone nasal spray, albuterol metered dose inhaler, potassium bicarbonate supplements, omeprazole capsules, alprazolam, and analgesics like tizanidine hydrochloride and oxycodone hydrochloride, which were all continued throughout the admission period. Coreg and amiodarone were restarted on day 2 once her blood pressure improved. The patient was discharged about a week after admission and was reviewed in the outpatient department later with no evidence of ongoing muscle damage.

## 3. Discussion

In this case report, we describe sacubitril/valsartan as the sole cause of rhabdomyolysis which is a rare adverse drug event. The patient presented with the two cardinal features of rhabdomyolysis namely muscle weakness and dark colored urine which subsequently led to hyperthermia and acute renal failure. In our patient, we considered two triggers which are the possibility of an infection and the new drug. Infection as a possibility was ruled out with no localizing symptoms, inability to isolate an organism on cultures, and an almost immediate resolution of the leukocytosis after the dehydration was corrected. The possibility of viral infection was unlikely given the complete blood count results which had neutrophilic predominance. This led us to conclude that sacubitril/valsartan is most likely responsible for the rhabdomyolysis exhibited by this patient.

There is a clear temporal association with de-challenge positive making it a “probable” cause of the adverse event observed as per the World Health Organization causality assessment [8]. The total score obtained in the Naranjo’s algorithm was 6 which classifies the causality as probable once again [9]. According to the Schumock and Thornton preventability scale, the ADR is not preventable and based on the Hartwig and Siegel Severity Assessment Scale, the severity of the reaction is placed at level 5 which involves withholding of the suspected drug and a stay at the intensive care unit [10,11]. The mechanism of how sacubitril/valsartan causes rhabdomyolysis is not clear.

## 4. Conclusions

We report a rare adverse event likely caused by sacubitril/valsartan. However, further research needs to be done to answer the exact mechanism of how it causes muscle damage. We also recommend that baseline CPK, LDH, and AST values be done for patients at high risk and if on any other concomitant medication that is known to cause muscle damage. We also encourage physicians to report similar adverse events that have already occurred or those which may occur in the future.

## Figures and Tables

**Table 1:** Laboratory investigations from day 1 to 7 of hospitalization.

	Day 1	Day 2	Day 3	Day 4	Day 5	Day 6	Day 7
Leucocytes (cu mm)	16.2	15.9	12.2	9.4	7.0	5.6	9.0
Erythrocytes (cu mm)	3.68	3.48	3.01	2.90	2.92	3.11	2.97
Hemoglobin (g/dL)	11.0	10.3	9.0	8.6	8.6	9.1	8.7
MCV (fL)	92.1	90.5	90.4	90.0	91.8	92.3	91.2
MCH (pg)	29.9	29.6	29.9	29.7	29.5	29.3	29.3
MCHC (g/dL)	32.4	32.7	33.1	33.0	32.1	31.7	32.1
RDW (%)	14.9	14.3	13.9	14.2	14.3	14.2	14.1
Platelets (K/UL)	309	300	253	273	277	238	288
Immature Gran (%)	0.2	0.3	0.2	0.4	1.4	1.3	1.7
Neutrophils (%)	85	92	89	85	58	72	65
Lymphocytes (%)	8	5	6	10	30	20	26
Monocytes (%)	7	4	5	5	10	7	8
Eosinophils (%)	0	0	0	0	0	0	0
Sodium (mmol/L)	134	138	138	139	141	141	145
Potassium (mmol/L)	4.6	3.6	3.1	2.6	2.6	3.8	3.5
Chloride (mmol/L)	100	102	104	106	110	109	113
Carbon Dioxide (mmol/L)	20	24	23	24	25	23	25
Whole Blood PCO_2_ (Vol %)	18.9						
Anion Gap	14.0	12.0	11.0	9.0	6.0	9.0	7.0
BUN (mg/dL)	44	38	36	32	31	25	24
Creatinine (mg/dL)	4.21	2.82	1.94	1.53	1.49	1.26	1.19
Est. GFR (African Amer.)	13	21	33	45	46	56	≥60
Glucose (mg/dL)	144	162	129	121	102	122	125
Hemoglobin Alc (%)		6.1					
Lactic Acid (mmol/L)	1.4	0.6					
Calcium (mg/dL)	7.9	8.1	8.0	8.1	7.8	8.0	8.2
Phosphorous (mg/dL)		4.1	2.6	2.2	2.2		
Magnesium (mg/dL)		1.9	1.8	1.7	2.0	1.6	1.6
Total Bilirubin (mg/dL)	1.38	1.45		0.61	0.33	0.43	
Direct Bilirubin (mg/dL)				<0.10	<0.10	0.12	
AST (IU/L)	376	205		132	84	63	
ALT (IU/L)	54	49		40	34	33	
Alkaline Phosphatase (IU/L)	72	86		57	46	53	
Creatine Kinase (IU/L)	61,453	43,236	18,773	9826	5451	2805	1512
CK-MB Fraction (ng/mL)	124.5						
Troponin I (ng/mL)	0.05						
BNP (pg/mL)	120						
Total protein (g/dL)	6.6	7.2		6.1	5.7	5.9	
Albumin (g/dL)	2.7	2.6	2.4	2.3	2.2	2.4	
Globulin (g/dL)	3.9	4.6		3.8	3.5	3.5	
Albumin/Globulin ratio	0.7	0.6		0.6	0.6	0.7	
Triglycerides (mg/dL)		267					
INR	1.0	1.0					
APTT (seconds)	32	34.2					

MCV—Mean Corpuscular Volume; MCH—Mean Corpuscular Hemoglobin; MCHC—Mean Corpuscular Hemoglobin Concentration; RDW—Red cell distribution width; GFR—Glomerular Filtration Rate; AST—aspartate transaminase; ALT—Alanine Transaminase; CK-MB—Creatinine Kinase—Muscle Brain; INR—international normalized ratio; APTT—activated partial thromboplastin time; BNP—B-Natriuretic Peptide; BUN—Blood urea nitrogen.
